# Comparative effectiveness of antiviral therapies in chronic hepatitis B and C management

**DOI:** 10.6026/973206300221367

**Published:** 2026-03-31

**Authors:** Ashis Tiwari, Praveen Kumar Tagore, Sukh Dayal Kumhar, Abha Bardiya

**Affiliations:** 1 Department of General Medicine, Government Medical College, Datia, Madhya Pradesh, India; 2 Department of Anaesthesiology, Shyam Shah Medical College, Rewa, Madhya Pradesh, India

**Keywords:** Chronic hepatitis B, chronic hepatitis C, antiviral therapy, direct-acting antivirals, nucleoside analogues, sustained virologic response, treatment effectiveness

## Abstract

Chronic hepatitis B and C infections affect over 350 million people worldwide, leading to progressive liver disease that demands effective antiviral therapy. Hence, this study compares four antiviral regimens across 480 patients (4 groups of 120 each): tenofovir or entecavir for HBV and sofosbuvir-based or glecaprevir/pibrentasvir combinations for HCV over 48 weeks. Glecaprevir/pibrentasvir achieved superior HCV suppression (96.7% SVR versus 94.2% sofosbuvir-based, p=0.032), while HBV showed 100% tenofovir versus 87.8% entecavir suppression (p=0.421). Both drug classes demonstrated high efficacy in halting viral replication and minimizing progression to chronic liver damage. These results advance therapeutic knowledge by identifying glecaprevir/pibrentasvir as a marginally superior HCV regimen and reinforcing evidence-based antiviral selection for durable viral suppression.

## Background:

Chronic viral hepatitis is one of the greatest challenges of infectious diseases in the world, as hepatitis B virus (HBV) and hepatitis C virus (HCV) infections are estimated to impact 296 million and 58 million people in the world, respectively [1]. Not only does the burden of chronic viral hepatitis have an impact on the direct health effect, but it also has an impact on the number of deaths each year, estimated at 1.1 million, which is the aetiology of hepatocellular carcinoma and end-stage liver disease, which will be the focus of the transplant [2]. Although there has been high progress in the development of antiviral therapy in the last 20 years, the optimal approach to treatment choice and sequencing is still the subject of clinical studies and discussion [3]. Clinical management of chronic HBV has changed considerably since it was introduced as an interferon-based regimen and today the clinical recommendation is nucleoside and nucleotide analogues as a first-line treatment in the majority of patients [4]. Entecavir and tenofovir disoprox fumarate have become the favoured agents because of a high barrier to resistance and high viral suppression that is observed in patients who are treatment naive after 48 weeks of therapy [5]. Recently, an analogue tenofovir alafenamide has been introduced that has a better safety profile, especially in bone and renal toxicity safety concerns that have been expressed by tenofovir disoproxil fumarate [6]. Nevertheless, the comparative efficacy of these agents with different categories of patients and the most appropriate duration of treatment are still issues that should be examined. With the evolution of the direct-acting antivirals (DAAs), the chronic hepatitis C treatment paradigm has undergone revolutionary change, with the interferon-based regimens being replaced by a more effective and tolerable regimen [7]. Existing DAA combinations, including sofosbuvir-based regimens as well as newer pangenotypic combinations, including glecaprevir/pibrentasvir, have shown sustained virologic response rates of most populations in the range of over 95% [8]. Hepatitis C has become a potentially curable condition for most patients due to the introduction of shorter treatment regimens, less intricate monitoring needs and enhanced adverse event profiles [9]. Despite these breakthroughs in therapy, there are still some clinical issues in the treatment of chronic viral hepatitis. The treatment choices will have to take into consideration the patient-specific factors such as genotype, history of previous therapy, presence of cirrhosis, renal functioning and possible drug interactions [10]. Also, there are few published data on the real-world effectiveness of contemporary antiviral regimens in various patient groups and currently, the majority of studies are based on a selected group of clinical trials that might not be related to actual practice in clinical settings [11]. The resistance-related replacement, especially among patients who have undergone treatment for hepatitis C, has brought into light the need to select optimal initial therapy to maximise the success of treatment [12]. Economic factors also have a dominant place in the choice of treatment and there are considerable differences in prices between the available antiviral regimens that pose difficulties to health systems and to a specific patient [13]. The introduction of generic formulations and negotiated pricing deals has enhanced access to DAA therapy worldwide, yet an imbalance in the availability of treatments still persists, especially in resource-constrained environments [14].

Knowledge of the relative cost-efficiency of various antiviral strategies is needed to inform evidence-based guidelines in the treatment and health care policy choices. Recent systematic reviews and meta-analyses have tried to combine evidence available which compares the effects of antiviral therapies; however, most have been hindered by heterogeneity of study populations, regimens and measures of outcome [15]. The speed at which the therapeutic field is changing has meant that, relatively few head-to- head comparative data exist to support newer antiviral combinations; indirect comparisons and real-world evidence development have been required to inform clinical decision-making [16]. Moreover, little long-term safety and efficacy data exists about newer agents, which makes it uncertain as to how patients who need longer treatment should receive therapy or those with certain comorbidities [17]. Integration of patient-reported outcomes and quality of life measurement into comparative effectiveness studies has become accepted as a significant element of full treatment examination [18]. Conventional clinical outcome measures like viral suppression and biochemical response, though clinically meaningful, might not be able to capture the patient experience and treatment burden that comes with various antiviral regimens [19]. The effect of treatment on symptom severity, functional status and overall quality of life is important to understand so as to establish patient-based treatment methods and maximise therapeutic outcomes [20]. There are current evidence gaps in that there is very little comparative treatment effectiveness data in some special groups, including patients with decompensated cirrhosis, patients with substantial comorbidities and those who have failed previous treatment [21]. Also, best practices of treatment monitoring, treatment length and the best use of post-treatment monitoring would be investigated to achieve the best clinical outcomes at a minimum of healthcare resource expenditure [22]. The possibility of drug-drug interactions, especially in patients on a complicated medication regimen, is another domain that needs to be systematically assessed to provide treatment safety and efficacy [24]. Therefore, it is of interest to assess the relative efficiency of existing antiviral treatment in the management of chronic hepatitis B and C infections, with special consideration of the viral suppressive percentages, biochemical response, treatment tolerance and patient outcomes, considering various therapeutic regimens in clinical practice.

## Materials and Methods:

## Study design and setting:

It is a prospective, multicenter, observational cohort study that was undertaken in four tertiary care hepatology centres between January 2022 and December 2023.

## Population and sample size of the study:

The population I needed was a population of adult patients with chronic hepatitis B or chronic hepatitis C who were starting antiviral treatment. The calculation of the sample size was made regarding the predicted variation in sustained virologic response rates between the treatment groups. Taking a baseline response rate of 90 per cent of the known regimens, a difference of 5 per cent across treatment groups, 80 per cent power and 5 per cent level of significance, at least 200 patients per type of hepatitis were required. We planned to enrol 240 patients in each hepatitis cohort, making 480 patients to cover the possible dropouts and to enable us to make subgroup analyses.

## Inclusion and Exclusion criteria:

The inclusion criteria included patients with chronic hepatitis B or C infection verified using positive hepatitis B surface antigen or hepatitis C antibody with detectable levels of HCV RNA for at least 6 months, naive to antiviral therapy and compensated liver disease with Child-Pugh class A or B cirrhosis. Informed consent was obtained and the patients proved to be capable of meeting study procedures and follow-up requirements. The exclusion criteria were decompensated cirrhosis (Child-Pugh class C), active hepatocellular carcinoma or other malignancy, hepatitis D virus coinfection, HIV, or other hepatotropic viruses, severe renal failure (estimated glomerular filtration rate <30 mL/min/1.73m2), pregnancy or breastfeeding, recent history of active substance abuse within 12 months and concomitant use of medications with known major interactions with study antiviral regimens.

## Treatment groups and regimens:

Patients with hepatitis B were divided into two main treatment groups according to the selection of physicians and patient characteristics: tenofovir disoproxil fumarate 300 mg/day or entecavir 0.5 mg/day (1.0 mg/day in the case of lamivudine-resistant patients). The hepatitis C patients were grouped into two major treatment groups to include a combination of sofosbuvir (400 mg per day) with ledipasvir (90 mg per day) or velpatasvir (100 mg per day), depending on genotypes and cirrhosis conditions.

## Data collection and follow-up schedule:

Demographic, medical history, hepatitis infection characteristics, laboratory parameters, imaging studies and quality of life questionnaires were identified as baseline assessments. The baseline and week 4, 12, 24 and 48 laboratory monitoring was done in hepatitis B and hepatitis C patients, respectively, with further follow-up in week 12 and 24 following the completion of the treatment.

## Laboratory assessments:

Real-time polymerase chain reaction assays were used to measure the viral load and the lower limits of quantification used were 20 IU /mL of HBV DNA and 12 IU /mL of HCV RNA. Biochemical tests involved alanine aminotransferase (ALT), aspartate aminotransferase (AST), total bilirubin, albumin, international normalised ratio and estimated glomerular filtration rate. The patients with hepatitis B received further observation on HBeAg/anti-HBe status and hepatitis B surface antigen titer.

## Primary and secondary outcomes:

The most significant outcomes were viral suppression (undetectable viral load) at week 48 in hepatitis B (HBV DNA <20 IU/mL or HCV RNA <12 IU/mL) and sustained virologic response (undetectable viral load) at week 48 in hepatitis C (undetectable HCV RNA). Secondary outcomes were biochemical response (ALT normalisation), adherence to treatment, which was measured by pill count and patient self-report and health-related quality of life.

## Safety monitoring:

Safety assessment involved clinical, laboratory and adverse event reporting of the study at every study visit. Critical adverse events were reported to the coordinating centre and regulatory bodies within 24 hours, as it is required. Aggregate safety data was reviewed after every six months during the study period by an independent data safety monitoring board.

## Statistical analysis:

The SPSS version 28.0 software was used to conduct the statistical analyses. The continuous variables were evaluated using mean and standard deviation or median and interquartile range based on the normality of the distribution measured using Shapirowilk test. The categorical variables were described in frequencies and percentages. The independent t-tests or Mann-Whitney U tests were used when comparing the means of continuous variables between groups and the chi-square or Fisher's exact tests when comparing the means of categorical variables. Kaplan-Meier survival curves and log-rank tests were used to test the time-to-event analyses. Multivariate logistic regression was applied to determine how variables predicted treatment response and controlled for individual factors such as age, gender, genotype, baseline viral load and cirrhosis at baseline. All tests of significance were two-sided tests with a level of significance of p < 0.05.

## Results:

Among Hepatitis B patients, the mean age was comparable between the Tenofovir (51.8 ± 13.2 years) and Entecavir (53.0 ± 12.4 years) groups. Similarly, Hepatitis C patients treated with SOF-based therapy (55.1 ± 12.3 years) and GLE/PIB (54.3 ± 11.5 years) showed similar age distributions. Male predominance was observed across all groups, ranging from 56.7% to 61.7%. The mean BMI was slightly higher among Hepatitis C patients (28.1 ± 5.2 kg/m^2^ in SOF-based therapy) compared to Hepatitis B groups. Comorbid conditions such as diabetes mellitus and hypertension were more common in Hepatitis C patients (diabetes 23.3-25.8%, hypertension 35.0-37.5%) than in Hepatitis B patients (diabetes 15.0-18.3%, hypertension 28.3-31.7%). The prevalence of cirrhosis ranged from 23.3% to 30.0%, with slightly higher rates in the SOF-based group. Prior antiviral treatment was also somewhat more frequent among Hepatitis C patients (17.5-20.0%) compared with Hepatitis B groups (12.5-15.0%). Laboratory parameters showed similar baseline viral loads, ranging from 6.2 to 6.9 log^10^ IU/mL. ALT levels were moderately elevated across all groups, indicating active liver inflammation. Additionally, renal function (eGFR) was generally preserved and comparable among all treatment groups (85.9-89.2 mL/min/1.73 m^2^) (Table 1 - see PDF). For hepatitis C patients, sustained virologic response rates were achieved in 113/120 (94.2%) patients receiving sofosbuvir-based regimens compared to 116/120 (96.7%) patients receiving glecaprevir/pibrentasvir (p = 0.032). Among hepatitis B patients, viral suppression at week 48 was achieved in 107/120 (89.2%) patients receiving tenofovir and 105/120 (87.5%) patients receiving entecavir (p = 0.421). Treatment-emergent adverse events were generally mild to moderate in severity across all treatment groups. Gastrointestinal symptoms were the most commonly reported adverse events in hepatitis C patients, while renal and bone-related monitoring was more relevant for hepatitis B patients receiving tenofovir. Treatment completion rates were high across all groups, with 114/120 (95.0%) hepatitis B patients completing 48 weeks of therapy in both treatment arms. Among hepatitis C patients, treatment completion rates were 117/120 (97.5%) for sofosbuvir-based regimens and 118/120 (98.3%) for glecaprevir/pibrentasvir. The most common reasons for treatment discontinuation included patient preference, adverse events and loss to follow-up. Among patients with cirrhosis, viral suppression rates remained high but were slightly lower compared to non-cirrhotic patients. In hepatitis C patients with cirrhosis, SVR rates were 91.7% (33/36) with sofosbuvir- based regimens and 93.9% (31/33) with glecaprevir/pibrentasvir. Hepatitis B patients with cirrhosis achieved viral suppression rates of 85.7% (24/28) with tenofovir and 83.9% (26/31) with entecavir. Treatment-experienced patients demonstrated lower response rates compared to treatment-naive patients, particularly in the hepatitis C cohort, where prior DAA exposure was associated with reduced SVR rates (Table 2, 3 - see PDF). Multivariable logistic regression analysis identified several baseline factors associated with treatment response. For hepatitis C patients, higher baseline viral load (OR 0.82, 95% CI 0.69-0.97, p = 0.024) and presence of cirrhosis (OR 0.68, 95% CI 0. 47-0.98, p = 0.041) were associated with lower SVR rates. In hepatitis B patients, higher baseline HBV DNA levels (OR 0.79, 95% CI 0.64-0.98, p = 0.032) and HBeAg positivity (OR 0.71, 95% CI 0.52-0.96, p = 0.028) were predictive of lower viral suppression rates.

## Discussion:

The study is a comprehensive prospective study with pertinent real-life evidence of comparative effectiveness of the modern-day antiviral treatment against chronic Hepatitis B and C infection. The results indicate a high percentage of overall response rates in all the treatment regimens and certain differences in efficacy and safety profiles that can be used in clinical decision-making and treatment optimisation strategies. The statistically significant yet clinically insignificant difference between the superior virologic response rates with glecaprevir/pibrentasvir versus sofosbuvir-based regimens (96.7% versus 94.2) is a statistically significant but clinically insignificant difference that is consistent with recent real-world findings that have shown the high effectiveness of pangenotypic DAA combinations [24]. These data correlate with those of registration trials and later cohort studies in the real world that have affirmed glecaprevir/pibrentasvir as an extremely successful treatment alternative in all types of hepatitis C [25]. The high SVR rates with both regimens support the revolutionary role of DAA therapy in the treatment outcome of hepatitis C and justify the current guidelines that recommend the regimens as first-line therapy in the majority of patients [7]. The similar viral suppression rates between tenofovir and entecavir in HB patients (89.2% versus 87.5) are in line with similar head to head studies and meta-analyses of these first-line nucleos (t) ide analogues that have shown similar efficacy [26]. These results can be used in work with the existing clinical practice guidelines, which suggest using both agents as the first-line therapy and the choice of the treatment is not to be made based on clinical considerations but rather depends on medical factors (including renal function, bone health, drug interactions, etc.) [27]. High rates of overall response in this study were observed, which is attributable to the high potency of modern nucleoside analogues and the significance of these agents in achieving viral suppression in the long term [28]. The safety profiles noted in this study were mostly in line with reported patterns of adverse events associated with each of the therapeutic classes. The greater percentage of renal function deterioration and bone density reduction with tenofovir than entecavir (6.7% versus 1.7% and 5.0 versus 0.8, respectively) is an indicator of already established safety issues with tenofovir disoproxil fumarate [29]. The results obtained highlight the necessity of baseline and continued monitoring of renal and bone conditions among patients undergoing tenofovir-based therapy, especially in elderly patients and those with pre-existing risk factors [30]. The use of tenofovir alafenamide in the form of a substitution with a better safety profile offers more treatment options to the patients at risk of getting these complications [31]. The high tolerability rate of DAA regimens in the present study, with fewer cases of treatment discontinuation and mostly mild adverse events, is a huge improvement compared to previous treatments that were based on interferon-based treatments [32]. The comparable adverse events using the sofosbuvir-based combinations and glecaprevir/pibrentasvir favour the application of either of the regimens based on tolerability and the choice of drug is mainly based on efficacy, drug interactions and patient characteristics [8]. The fact that treatment completion rates have been high with all regimens of DAA proves that it is possible to cure the vast majority of patients with hepatitis C using modern therapy [33]. The subgroup analyses that indicate the decreased response rates among patients with cirrhosis and previous exposure to treatment procedures outline several clinical implications for treatment planning and counselling of patients.

Comparable percentages of SVR in cirrhotic patients, though again above 90 per cent with both DAA regimens, underscore the need to treat them as soon as they develop hepatitis B [34]. These results confirm existing proposals of treating all patients with chronic hepatitis C infection irrespective of the disease progression stage and recognise that treatment efficacy can be maximised when it is given to patients without progressed fibrosis [35]. This finding of markers of baseline viral load and severity of disease to predict response to treatment gives clinically relevant data in the management of patients and monitoring of their treatment response. In hepatitis B and C, a higher baseline viral load was linked to the reduction in the response rate, indicating that these groups of patients might require closer monitoring or other methods of treatment [36]. The correlation between the positive HBeAg and the decreased viral suppression in patients with hepatitis B indicates the increased difficulty in the viral suppression process in patients with high replicative activity [37]. The apparent quality of life improvement in all of the treatment groups simplifies the overall advantages of effective antiviral treatment that go beyond virologic and biochemical results. Those marked changes, especially in hepatitis C patients, are probably due to the shorter duration of DAA therapy and the curative effect of the treatment in contrast to the unlimited treatment of hepatitis B [38]. These PRMs give valuable evidence in favour of the benefit of the use of antiviral therapy from the patient perspective and could potentially affect the acceptance and adherence to the treatment [21]. Several limitations of the study should be considered when analysing these findings. Although the observational study design offers rich real-life evidence, it is the source of the risk of confounding factors that could affect the treatment outcomes, regardless of the statistical adjustments. The comparatively shorter follow-up, especially on hepatitis B patients, limits the evaluation of the long-term treatment efficacy, as well as the sustainability of viral control [39]. Also, the population of the study was mainly comprised of patients attending special hepatology centres, which might not be a complete picture of the treatment outcomes in the primary care or neighbourhood practice [40]. Although the economic impact of treatment choice was not specifically measured in this paper, it is a significant factor that healthcare systems and individual patients need to take into account. High prices of different antiviral regimens, especially of the DAA therapy, pose obstacles to both access and sustainability of treatment [41]. In future studies, the systematic economic analysis must be involved to enhance the value-based treatment decision-making and resource allocation optimisation [42]. Cost barriers can be overcome by treating the world through the development of generic formulations and bio similar substitutes [14]. The development of resistance- related replacements, although being rare with modern antiviral treatment, is also something that should be monitored and studied continuously. The genetic barrier to resistance of both the first-line treatment of hepatitis B and C is high and has therefore significantly lessened the clinical effect of resistance with regard to the previous period of treatment [43]. Nevertheless, ongoing surveillance of patterns of resistance and formulation of salvage treatment to failures of treatment are still significant in the best management of the patients [44]. The future direction of research should be on streamlining treatment plans for special populations, such as patients with decompensated cirrhosis, serious comorbidities and those with multimodal drug regimens that can raise the possibility of drug interactions [21]. Also, research on shorter therapy time, especially in the case of hepatitis C patients who are at low risk of treatment failure, could also help to enhance treatment convenience and lower costs without compromising the cure rates [45]. Combinations of prevention measures that will be developed, including vaccination, screening and treatment measures, will be fundamental in the realisation of global hepatitis elimination objectives [46].

## Conclusion:

Modern antiviral therapies for chronic hepatitis B and C achieve high viral suppression rates with excellent safety and tolerability, confirming guideline recommendations. Glecaprevir/pibrentasvir outperforms sofosbuvir-based regimens for HCV; tenofovir and entecavir show equivalent HBV efficacy, with tenofovir requiring renal/bone monitoring. These findings streamline clinical decision-making toward viral hepatitis eradication, warranting future research on special populations, long-term outcomes and cost-effectiveness.
